# Clinician perceptions of a novel metric to compare Veterans Health Administration hospitals on antibiotic prescribing at hospital discharge

**DOI:** 10.1017/ash.2026.10842

**Published:** 2026-09-01

**Authors:** Stacey Hockett Sherlock, Brittany L. Anderson, Nicole L. Johnson, Kimberly C. Dukes, Daniel J. Livorsi

**Affiliations:** 1 Center for Access and Delivery Research and Evaluation (CADRE), Iowa City Veterans Affairs (VA) Healthcare System, Iowa City, IA, USA; 2 Department of Internal Medicine, University of Iowa Carver College of Medicine, Iowa City, IA, USA

## Abstract

Antibiotic overuse at hospital discharge is common and often overlooked. We conducted interviews with 91 clinicians across 9 Veterans Health Administration hospitals to assess perceptions of a metric comparing discharge antibiotic-prescribing. Clinicians found the metric valid and meaningful, but successful implementation will require more granular contextual data and quality-improvement framing.

## Introduction

Antibiotic stewardship (ASP) programs in hospital settings are generally oriented toward the inpatient environment.^1^ However, antibiotic overuse is common at hospital discharge.^2,3^ ASP metrics that capture antibiotic-prescribing at discharge can identify opportunities to optimize prescribing.^4^ We conducted a qualitative analysis of clinician perceptions of a novel risk-adjusted metric that compares the frequency and duration of oral antibiotics prescribed at discharge at Veterans Health Administration (VHA) hospitals. Details on metric development, validation, and included variables were previously published. ^5^ This analysis is a subset of a study evaluating optimization of antibiotic prescribing at discharge.

## Methods

### Data collection

Among 66 VHA hospitals classified as high-complexity by the VHA’s Healthcare Analysis and Information Group, we recruited 11 drawn from three performance quadrants of the metric. The fourth quadrant, representing more frequent, shorter duration prescriptions, was not included because it comprised largely lower-complexity hospitals. Each facility’s ASP pharmacist was invited to an informational call, which included a brief interview about their facility’s stewardship processes. If the site decided to participate, our team collaborated with each facility’s ASP team to decide if an in-person or virtual site visit was a better fit for conducting interviews. The ASP teams provided contact information for clinicians involved with prescribing and/or monitoring antibiotic use at discharge. Clinicians were recruited by email and were typically scheduled for interviews with two qualitative team members. From July 2024 to June 2025, we conducted interviews with ASP physicians, ASP pharmacists, clinical pharmacists, hospitalists and other providers, and laboratory staff. The study was approved by the University of Iowa Institutional Review Board (#202311339) and the Research and Development Committee at the Iowa City VA.

**Figure 1.**
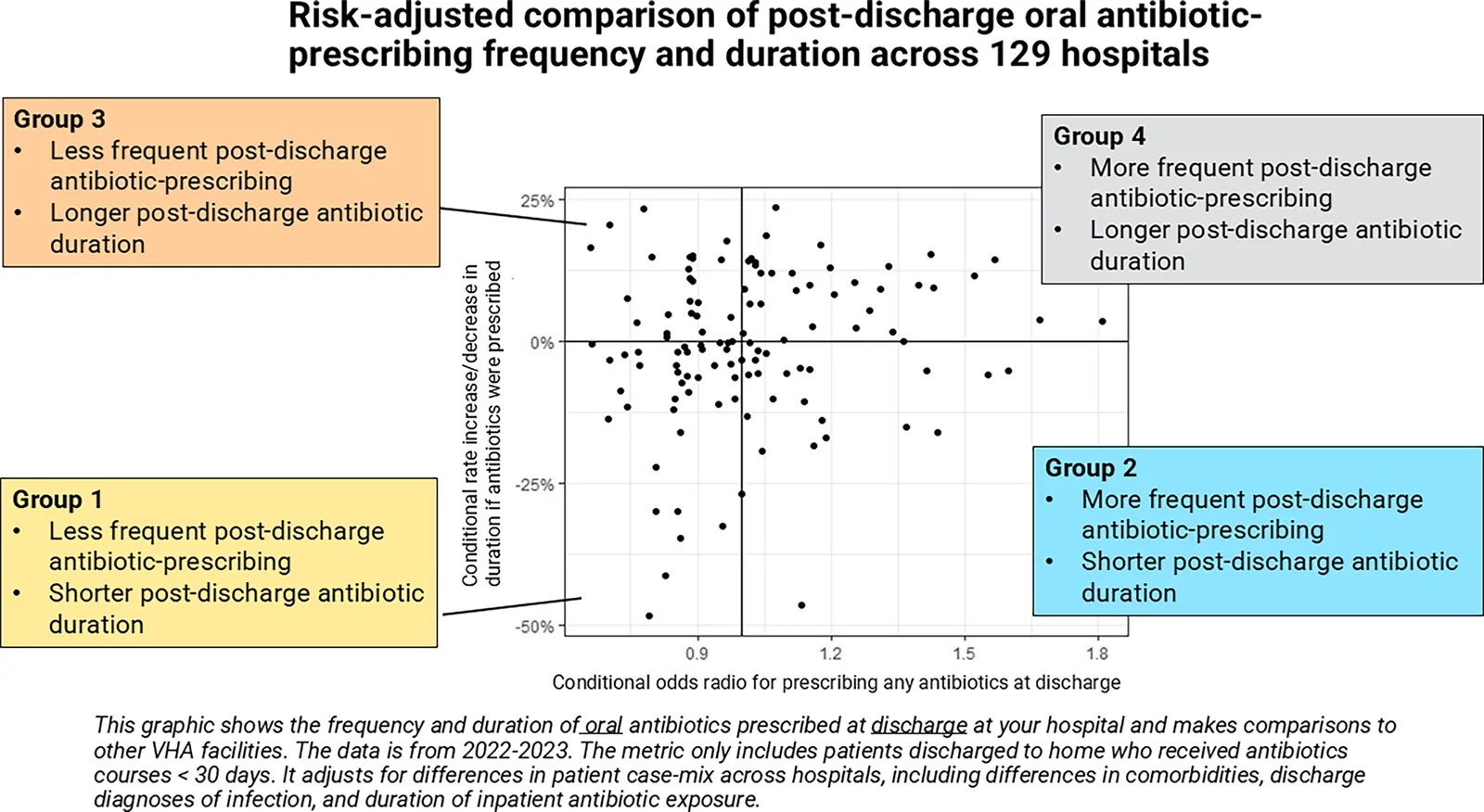
Descriptive graphic showing the risk-adjusted comparison of post-discharge oral antibiotic-prescribing frequency and duration, including Group specifications and description of the metric data and its development, as provided in interviews.

The interview guide was designed to evaluate understanding and perceptions of the usefulness, acceptability, and validity of the metric. During the interview, each participant was shown a graph of their facility’s performance on the metric compared with the performance of the anonymized population of all other VHA facilities (Figure 1). Each participant was also given a brief description of the metric data and its development. The qualitative team (SHS, KCD, NLJ, BLA) conducted and recorded in-person or MS Teams interviews with participant consent. Interviews averaged about 30 minutes. Interviews were transcribed and we imported audited transcripts into qualitative analysis software, MAXQDA (VERBI Software, 2026).

### Data analysis

Each interview transcript was coded by team members who met to determine consensus. The team used both deductive coding based on the interview guide and emergent inductive thematic coding.^6^

## Results

Of 11 invited sites, 9 geographically distributed sites participated and were categorized by postdischarge antibiotic prescribing frequency and duration: Group 1 (less frequent, shorter duration), 3 sites; Group 3 (less frequent, longer duration), 4 sites; and Group 4 (more frequent, longer duration), 2 sites. We conducted 91 semi-structured interviews across 3 in-person (n = 25) and 6 virtual (n = 66) site visits.

### Response to metric

Clinicians across performance groups were receptive to the metric and consistently identified modifications they viewed as necessary for its effective use as a stewardship measure. Table 1 provides exemplar quotes illustrating selected themes.

**Table 1.** Exemplar quotes for selected themes; lightly edited for readability Table 1 long description.

Face validity	
Comparison to other VAs is useful	“I feel like you have to…do some sort of comparison to see where you fall. And not everything is perfect, right? But I feel like you have to start somewhere.” *Pharmacist Site B* “I think it’s a fair way to look at hospitals. I mean, there has to be some way to tell people if they’re, you know, prescribing more than a partner hospital. So, I guess what better way to do than this chart?” *Pharmacist Site F* “I actually think it’s really helpful to see this [metric]. I like knowing, you know, how we do as a site compared to the national group, and that’s one of the benefits of being in the VA.” *Physician Site I*
Stewardship utility	
Practice does not always align with guidelines	“Despite the fact that there’s generalized agreement in terms of the use of antibiotics and the length of use of antibiotics and the type of antibiotics to use and the term antibiotic stewardship, there’s a lot of give and take.” *Physician Site B* “In an ideal world, yes, I mean it would be great if everyone kind of was accountable, but in real world I think it would be pretty hard to implement and maybe maintain something like this. Because I think you could say that with any stewardship intervention, because it’s ultimately up to the physicians who are managing the patient and who know the patient and feel comfortable discharging the patient on more or less antibiotics. It’s hard to enforce them to directly do shorter therapies. And then there’s the fear that goes along with possibly under-prescribing and things like that.” *ASP Pharmacist Site A*
Potential for stewardship usefulness	“I’m a big believer in feedback to providers to kind of help nudge them in a direction. I think that this type of information could be useful. I think without a little bit more details, it would maybe be, it would be met with a lot of questions and maybe a little resistance.” *Physician Site C* “It’s just like something good also to show because nowadays in stewardship they want a lot of metrics. What we’re doing, how to show what we’re doing, and it’s so hard to find metrics to show them because we work on a lot of projects, but then it’s so hard to follow-up on them.” *ASP Pharmacist Site D* “It doesn’t necessarily…say that just because we have more frequent antibiotic use, that it’s inappropriate. Like, we can’t really tell that from this [metric], but it at least lets us know where we stand so that we can at least look into it.” *ASP Pharmacist Site F* “What I’ve come to learn is that when it comes to education, then really, I mean, everyone ought to see this. This is, anyone who’s prescribing antibiotics. Because that’s the best chance you’re gonna get that someone is gonna change their practice. And whether that’s 1% of the 10% or more.” *Physician Site G*
Additional needed data	“Some of the questions I’d want to know are, can we find out what service? Or what providers? Or what antibiotics are being overused, so that we can look at that in more detail? And then how many days are being prescribed?” *ASP Pharmacist Site B* “I don’t know if this is data that would be published or aggregated quarterly or monthly or whatever, right. But looking at those trends over time to see, what’s the pattern of prescribing, are these flips or are these trends and then I think getting into more specific, how can we target--, like are there certain types of infections where we are consistently kind of outside of the mean?” *Pharmacist Site H*
Fairness and potential for punitive use	
Metric cautions	“It’s hard to say, you know, and it [the data] could be based on kind of where places are [regionally]. Maybe they use a lot of community care for certain things and other--, There’s just so many variables…I wouldn’t say, oh, attach it to an incentive …So even if it’s information and it gives you a reason to think about it and maybe nudges you a certain way. I think that’s fine, but I think not using it for quality metrics” *Physician Site H* “I think that one of the questions my group would have if presented this data is, but do we have better outcomes? Because we might be prescribing more than other people, but that’s because we might be doing a better job than them…so we would probably want to see some correlation between this data and other quality data.” *Physician Site B* “I think everyone has differing resources and I think that…we are probably better situated than most of our counterparts, even in the VISN…So it’s not fair to compare in that regard. If you have dedicated stewardship resources…and your whole job is to just target discharge antibiotics, well, then that shifts the way you do your job and you may not have as many of us, but now you’re targeting one thing, so you should be effective…but if it’s two CPPs and a 200 bed hospital trying to put out fires and answer doctor questions, then you can’t expect them to meet the same metrics that work for us.” *Pharmacist Site E*

### Reaction to performance on the metric

Clinicians often proposed a potential explanation for why their facility was in a specific performance group. Those in Group 1, which prescribed postdischarge antibiotics less often and for shorter durations, frequently credited their metric performance to strong ASP programs and institutional stewardship culture. In contrast, those in Groups 3 and 4, which prescribed postdischarge antibiotics for longer durations, more often reflected on patient complexity or specific clinical services that were likely overusing antibiotics.

### Face validity

The majority of clinicians across performance groups found that the metric made sense and conveyed results clearly. Clinicians generally agreed that benchmarking discharge antibiotic prescribing against peer VHA facilities was a reasonable and informative approach. Some noted the value of being part of an integrated national healthcare system, which facilitates meaningful comparisons.

### Stewardship utility

Clinicians expressed that the metric could support stewardship practices by reinforcing appropriate prescribing and identifying areas for improvement. However, many emphasized that the metric alone was insufficient to drive targeted interventions. Clinicians highlighted that they needed additional granular data to inform effective interventions, including service-specific prescribing, which infections are being overtreated, and how close their hospital was to categorization in a different performance group. Across performance groups, clinicians requested more information about how the metric accounted for differences in patient case-mix. Clinicians indicated that access to details about the methodology would be important for building confidence in the metric and supporting its acceptance as a stewardship measure.

### Fairness and potential for punitive use

Perceptions of fairness varied across performance groups and clinician roles. Although many clinicians acknowledged the value of comparative benchmarking, some voiced concerns about how the metric might be applied in practice. A central concern was that comparative prescribing data could be misinterpreted as evidence of inappropriate care without sufficient consideration of clinical context or patient outcomes. Some were concerned about the possibility of the metric being used punitively or as a standalone quality measure without considering whether prescribing patterns aligned with appropriate antibiotic prescribing.

## Discussion

In this study, we found that clinicians considered a novel metric for comparing antibiotic prescribing at hospital discharge to be clear and clinically meaningful, suggesting that it may help hospitals identify opportunities for improved antibiotic stewardship at discharge. However, clinicians emphasized the need to be provided with granular data (eg, prescribing service) for the metric to be utilized. Clinicians also raised concerns about fairness and potential punitive use.

Discharge prescribing remains understudied compared to inpatient stewardship, despite evidence of frequent antibiotic overuse at discharge.^1–4^ Standard metrics for hospital-based ASP programs do not capture data on postdischarge antibiotic use, though prior work highlights the potential usefulness of a postdischarge antibiotic use metric. For example, an evaluation of a national inpatient stewardship initiative found that benchmarking was identified as an effective strategy because it provided performance context, motivated staff to improve, and guided targeted interventions; importantly, ongoing communication and education were needed to sustain engagement.^7^ Previous qualitative work within the VHA found that ASP leaders valued standardized, risk-adjusted benchmarking metrics for inpatient antimicrobial consumption but had concerns about the ability of risk adjustments to account for facility differences.^8^ Consistent with prior work,^9^ clinicians in our project emphasized that benchmarking would be most effective when framed as a quality improvement strategy rather than punitive or invasive. Addressing these concerns and clearly framing the metric as a tool for quality improvement will be essential for its successful implementation.

In practice, the ASP team could use their facility’s metric performance to determine whether further assessments of discharge antibiotics are warranted and whether additional stewardship strategies focused on discharge are needed. In a prior study across 7 hospitals, less antibiotic overuse at discharge was associated with high-quality pharmacist-physician interactions, infrastructure and knowledge to support stewardship, and highly engaged Infectious Disease physicians.^10^

Our study has limitations. Findings may not be generalizable beyond the VHA context. Although we interviewed personnel from only 9 sites, the 91 interviews included perspectives from both those leading stewardship efforts and those expected to adhere to them. A strength of the project is that there is little data on how hospital-level postdischarge stewardship metrics are perceived by end-users, making our findings an important contribution for future metric development and implementation efforts.

## Research transparency and reproducibility

Data not publicly available.

## Table 1. Long description

The table has four columns: Face validity, Stewardship utility, Potential for stewardship usefulness, and Additional needed data. It contains quotes from clinicians across different sites, categorized under these themes. Each row presents a unique quote, lightly edited for readability, reflecting the clinicians’ perspectives on the utility and necessary modifications of antibiotic stewardship metrics. The table includes quotes from various sites such as Pharmacist Site B, Physician Site F, and ASP Pharmacist Site A, highlighting the importance of feedback, the challenges in aligning practice with guidelines, and the need for additional data to improve stewardship efforts.

Navigate back to Table 1..
